# Dr. Drake, on the Digitalis in Tubercular Consumption

**Published:** 1799-10

**Authors:** Nathan Drake

**Affiliations:** Hadleigh


### *To the Editors of the Medical and Physical Journal.*

GENTLEMEN,

I HAVE had, ſince you laſt heard from me, three caſes of confirmed tubercular conſumption; two of theſe were terminating the ſecond ſtage, the third was advanced into the laſt. They were all exquiſitely marked inſtances of the diſeaſe. The firſt, a young man about twenty-eight years of age, had loſt a brother and a ſiſter by the complaint; he had been under the care of two phyſicians* in this neighbourhood previous to my being called in, but ſo rapid had been the progreſs of the ſymptoms, that, though not three months had elapſed ſince the commencement of the diſeaſe, the ſecond ſtage was far advanced, attended with copious expectoration, profuſe colliquative ſweats, and full-formed hectic; his pulſe near 120. I puſhed, in this caſe, the doſe of tincture to 120 drops in twenty-four hours, but ſlight delirium ſupervening, I reduced it again to 90. The pulſe one day ſunk to 68, but, in general, during the whole time he was under the influence of the digitalis, ſeldom fell below 68, and never, except one day when it ſuddenly mounted to 120, roſe above 90 †. The colliquative ſweats and febrile exacerbations greatly abated, but the expectoration ſtill continued purulent and copious, though not increaſing. It was only through the aſſiſtance of opium, that I was able to give ſo large a doſe of ſaturated tincture; it produced not much ſickneſs, but conſiderable vertigo, exceſſive languor, and ſuch univerſal torpor, that the patient, though ſitting up, was unwilling either to ſpeak or move. After being three weeks under the full influence of the digitalis, with the ſymptoms nearly ſtationary, he became averſe towards proſecuting it further, nor could I perſuade either him or his friends to permit a more extenſive trial. On relinquiſhing the fox-glove, his pulſe ſpeedily returned to the former ſtandard, and the other ſymptoms making their uſual progreſſion, he died in about five or ſix weeks after.

The ſecond patient had been ſuffering under the complaint for nearly two years, and was certainly in the laſt ſtage; he was greatly emaciated, with purulent expectoration, profuſe ſweats, and a pulſe between 120 and 130. By cautiouſly increaſing the doſe of tincture I was able to reduce his

* Dr. MACLEAN and Dr. CLUBBE, by whom the digitalis, I underſtand, had been adminiſtered in the form of powder; the wiſhed-for effect in conſumption, however, can ſeldom be produced by the powder, though in dropſy it ſeems preferable to any other mode of exhibition.

† His ſurgeon, Mr. SALTER, of Boxford, was very attentive, during my abſence, in noting the variations of his pulſe.

his pulfe to 80. Seventy drops, however, in twenty-four hours, formed the largeft dofe he could take; even with this quantity confiderable pain in the head and eyes, with vertigo and ficknefs, took place, and he refolutely refufed to continue the medicine. On taking my leave, I ordered the furgeon, Mr. Newell, of Colchefter, to repeat the fox-glove, if poffible, in a concealed way; this I underftand was done, but a return of vertigo and pain again put a ftop to the experiment. The expectoration was not diminifhed. He died about nine weeks after I laft faw him.

The third, a man of fifty-two, was terminating the fecond ftage when I was called in, and had lately loft a fifter by the complaint. His expectoration was very purulent, his colliquative fweats exceffive, and his hectic exacerbations ftrongly marked; cough and difficulty of breathing great; much emaciated, and his ftrength fo reduced, that he cannot but with extreme difficulty quit his bed for many minutes. He refides at Hadleigh, and was therefore immediately under the daily attention of myfelf and Mr. Bunn, an active and intelligent furgeon of this place, and who threw into a tabular form the variations of his pulfe, and the dofes of his tincture. He took the digitalis in infufion of quaffia for feveral weeks, and the tincture was gradually increafed to 100 drops per day. His pulfe was reduced from 120 to 50, and kept thus reduced for better than a fortnight, with little ficknefs, and with only flight attacks of vertigo. His expectoration rapidly decreafed; his colliquative fweats, cough and dyfpnœa gradually vanifhed; his appetite, which was fo impaired that a fmall quantity of broth oppreffed him, became keen; in fhort, the man is now in perfect health, and purfuing his ufual occupation.

Thus of five cafes of confirmed confumption (including thofe of Grimes and Marris), three have been perfectly recovered. I have no expectation, however, that upon a larger fcale, the proportion of fortunate to fatal refult would be what my experience has given.

Two cafes of cough, one of which was attended with evident purulent expectoration, and a cafe of vomica, have alfo occurred to me lately, and have been cured by the tincture of fox-glove, but in thefe no fymptoms of phthifical predifpofition were prefent.

It is fingular that I have never yet had an opportunity of exhibiting the digitalis in incipient tubercular confumption, that is, previous to any ulceration. A fatal delufion feems hitherto to have prevented an early application to medicine in this moft deftructive of all difeafes. As a proof, however, of what the digitalis can do, even in the very lateft period of this complaint, I produce the following account:—In March laft I was desired to visit Bridget Baker, a poor girl of this place, aged seventeen, whom I found apparently dying. She had been for some time gradually sinking under a confirmed phthisis; was reduced to a mere shadow, confined to her bed, and only moved from thence by assistants to another, until the former should be made. Pulse 140; breathing laborious and painful, cough almost incessant; expectoration mere pus, and with difficulty ejected; it appeared that a few hours might decide her fate. Being urged, however, by her mother, I began with a small dose of the tincture twice a day. It seemed to give relief, and now anxious that it should be afforded to its utmost extent, I gave her the tincture with my own hands during the whole of her illness, twice or thrice a day, slowly encreasing the dose, until she took 90 drops per day. Her pulse was reduced to 56; her expectoration much diminished; her cough, pain, and perspiration, greatly abated, and she took some wine and food daily. She lived near five weeks, and I regularly gave her the tincture twice or thrice a day, as circumstances warranted. She had no vertigo, not much sickness, nor much irregularity of pulse. She acquired little strength it is true, and never left her bed; but she was easy and tranquil during the day, and for the most part passed the night in sleep; her intellects were perfect to the last moment; her death was free from struggle and from pain, so gentle and imperceptible indeed, that the transition was with difficulty marked.

I am, &c.

Gentlemen,

Hadleigh, Your's most sincerely,

* *This interesting Letter was transmitted to us by a friend of Dr. Drake, so late that we received it only on the 18th of September.*

